# Patent Foramen Ovale Closure With Catheter-Directed Thrombolysis in Acute Pulmonary Embolism

**DOI:** 10.7759/cureus.83979

**Published:** 2025-05-12

**Authors:** Simran Koura, Amir Darki

**Affiliations:** 1 Internal Medicine, Loyola University Medical Center, Maywood, USA; 2 Cardiology, Loyola University Medical Center, Maywood, USA

**Keywords:** catheter-directed thrombolysis, patent foramen ovale (pfo), pulmonary embolism (pe), refractory hypoxemia, right ventricular dysfunction

## Abstract

Patients with acute pulmonary embolism (PE) often present with hypoxia; however, their oxygenation typically improves with appropriate respiratory support. Here, we discuss a case of persistent hypoxia in PE attributed to shunting through a patent foramen ovale (PFO). A 77-year-old woman with a history of hypertension and heart failure with preserved ejection fraction presented with acute shortness of breath. A CT angiogram demonstrated bilateral proximal PE involving the lobar pulmonary arteries extending into the segmental branches. Despite BiPAP ( (Bi-level Positive Airway Pressure) support, the patient had worsening hypoxia, with a partial pressure of oxygen (PaO_2_) of 57 mmHg. An echocardiogram demonstrated a pulmonary artery systolic pressure (PASP) of 44 mmHg with right ventricular systolic dysfunction and a right-to-left shunt secondary to a PFO. The patient underwent catheter-directed thrombolysis (CDT), resulting in PFO closure and improvement in PASP to 33 mmHg. Following treatment, she was successfully weaned off supplemental oxygen. Intractable hypoxemia due to a PFO in the setting of acute PE is a rare occurrence, with only a few cases reported. Previously, such cases have been treated with systemic thrombolysis or surgical embolectomy. To our knowledge, this is the first reported case in which CDT successfully resolved the hypoxemia and resulted in PFO closure. In patients with intractable hypoxia in the setting of acute PE, the presence of an interatrial shunt should be considered. CDT can effectively reduce pulmonary pressures by decreasing clot burden, which in turn may reverse the shunt and resolve hypoxia.

## Introduction

Acute pulmonary embolism (PE) is the third most common cause of cardiovascular death following myocardial infarction and stroke [1]. Dyspnea is one of the most common presentations of acute PE. Previous cases of acute PE have been reported in which severe hypoxemia was attributed to the presence of a patent foramen ovale (PFO). A PFO occurs when the foramen ovale, a structure present during fetal development in the interatrial septum, fails to close after birth [1]. PFOs are present in up to 35% of the general population and are generally asymptomatic [1]. However, the presence of a PFO can lead to paradoxical emboli in which a blood clot bypasses the pulmonary vasculature, thereby increasing the risk of stroke [1]. Additionally, in instances in which the pressures of the right side of the heart are greater than the left, PFOs can lead to right-to-left shunting resulting in deoxygenated blood being circulated to the body [1]. While patients with acute PE may present with hypoxemia, most patients would demonstrate a response to oxygen supplementation. However, in patients with a PFO in an acute PE, oxygen supplementation would not treat the underlying cause of hypoxemia and patients may remain persistently hypoxic. Here, we present a case of intractable hypoxemia in the setting of an acute PE secondary to a PFO, which resolved with catheter-directed thrombolysis (CDT).

## Case presentation

A 77-year-old woman with a history of heart failure with preserved ejection fraction and hypertension presented to the emergency department with acute-onset shortness of breath for the past day. Initial vitals were notable for an oxygen saturation of 93% on room air; the patient was otherwise hemodynamically stable. Initial laboratory results were notable for a brain natriuretic peptide of 189 (ref 1-100 PG/mL), a high-sensitivity troponin of 30 (ref 0-14 NG/L), an elevated D-Dimer of 14, 526 (ref <500 NG/mL), and a lactate of 1.8 (ref 0.5-1.6 mm/L).

**Figure 1:**
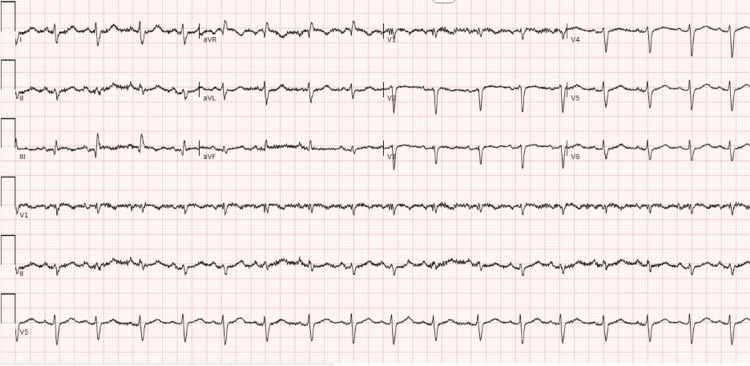
ECG at presentation demonstrating sinus tachycardia and S waves in lead I, and Q waves and T wave inversions in lead III.

Electrocardiography on presentation demonstrated sinus tachycardia with a rate of 105 and S1Q3T3 changes (Figure 1).

**Figure 2:**
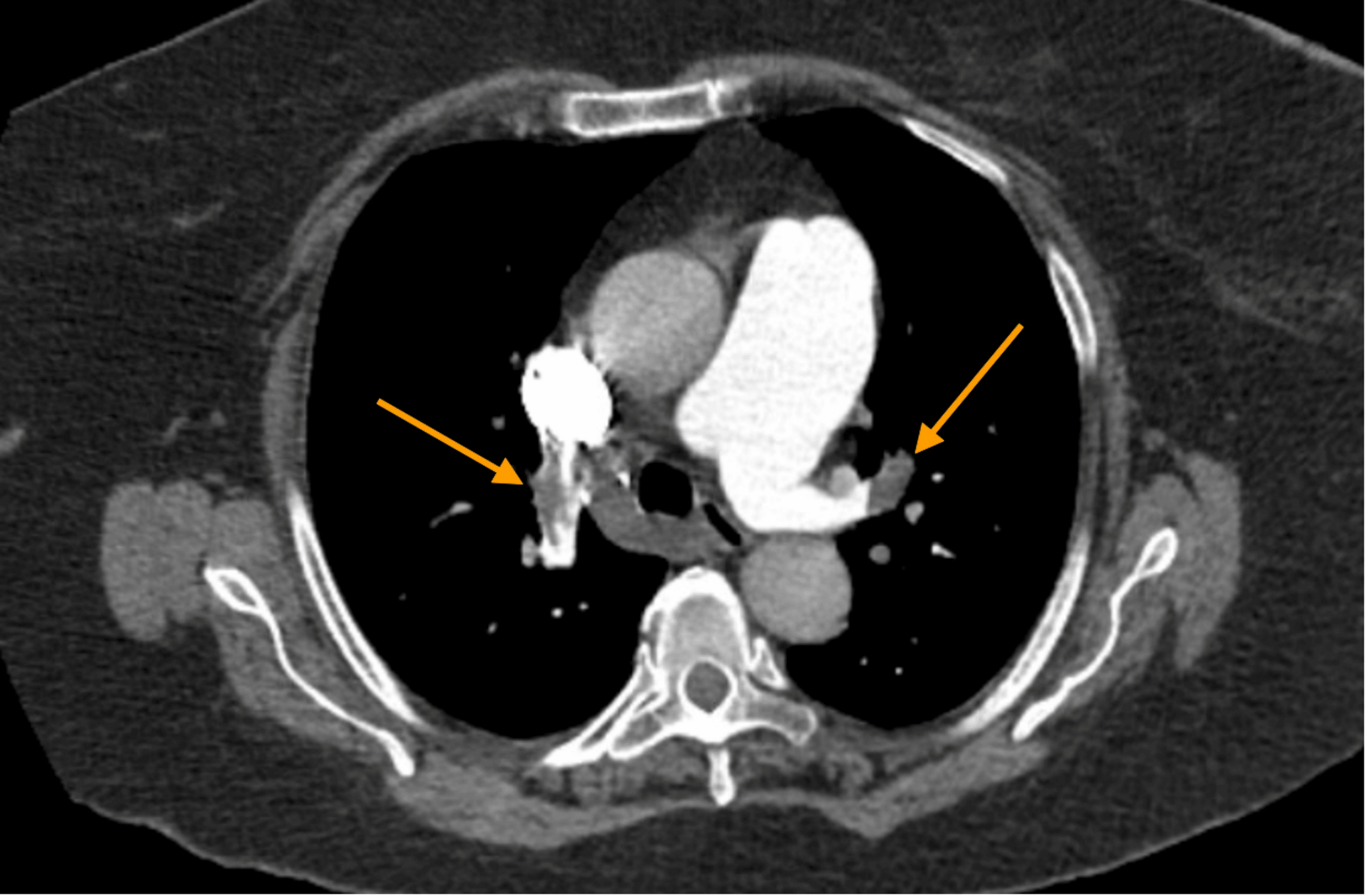
CT scan obtained at presentation demonstrating large proximal bilateral PE’s involving the lobar and segmental vessels. PE: pulmonary embolism.

A computed tomography pulmonary embolism (CTPE) revealed a large, proximal bilateral PE involving the lobar arteries and extending into the segmental vessels (Figure 2). Additionally, the pulmonary artery was dilated to 4.4 cm in diameter (ref 2.7 cm) and the right ventricle (RV) was more enlarged compared to the left ventricle (LV) with an RV/LV ratio of 1.28. Given evidence of RV ischemia, pressure overload on biomarkers, and the presence of RV dysfunction, the patient’s PE was classified as intermediate-high risk. She was immediately started on a heparin drip.

On day two of admission, the patient developed worsening hypoxemia with oxygen desaturation to 80% despite the use of a non-rebreather, prompting transfer to the intensive care unit. Arterial blood gas demonstrated persistent hypoxemia with a pH of 7.42, partial pressure of carbon dioxide (pCO_2)_ of 39 mmHg, and a PaO_2_ of 57 mmHg while on BiPAP. Echocardiography demonstrated significant RV enlargement with an RV/LV ratio of 1.3, pulmonary artery systolic pressure (PASP) of 44 mmHg, and a large right-to-left shunt secondary to a PFO (Table 1).

**Table 1:** Echocardiographic and catheter-obtained measures of right ventricular function before and after treatment with catheter-directed thrombolysis for the acute pulmonary embolism. CDT: catheter-directed thrombolysis.

Mechanism of measure	Parameter	Pre-CDT	Post-CDT	Reference range
Echocardiogram measures	Left ventricular ejection fraction	64%	60%	50-70%
	Right ventricle/left ventricle ratio	1.3	0.9	<1
	Pulmonary artery systolic pressure (mmHg)	44	33	<35
	Tricuspid annular plane systolic excursion (cm)	2.53	2.2	>1.7
	Right atrial pressure (mmHg)	8	Not obtained	0-5
	Right ventricle-right atrial pressure (mmHg)	36	30	10-25
	Shunt flow	Right to left	No longer present	Absent
Catheter-obtained measures	Pulmonary artery pressure (mmHg)	50/19 (35)	Right pulmonary artery: 37/11 (21). Left pulmonary artery: 40/13 (22)	<25/10
	Cardiac output (L/min)	6.28	Not obtained	4-8
	Cardiac index (L/min/m²)	2.86	3.00	2.5-4

**Figure 3:**
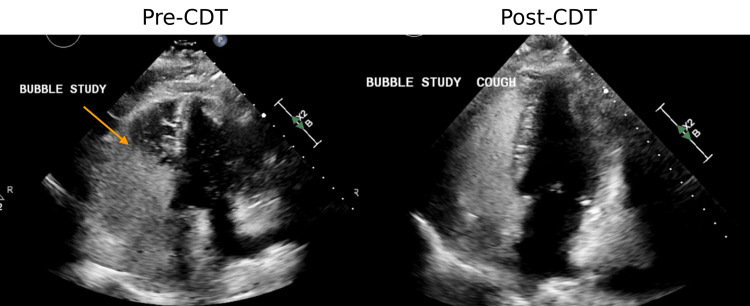
Pre-CDT (left), echocardiogram images demonstrated a positive bubble study indicative of a PFO. Following treatment of the acute PE with CDT (right), the bubble study was negative, suggestive of PFO closure. CDT: catheter-directed thrombolysis, PFO: patent foramen ovale, PE: pulmonary embolism.

Given the high clot burden, presence of RV dysfunction, and intractable hypoxemia, the patient underwent CDT. Following CDT, there was a significant improvement in the PASP to 33 mmHg, normalization of the RV/LV ratio at 0.9, and resolution of the right-to-left shunt (Table 1, Figure 3).

**Figure 4:**
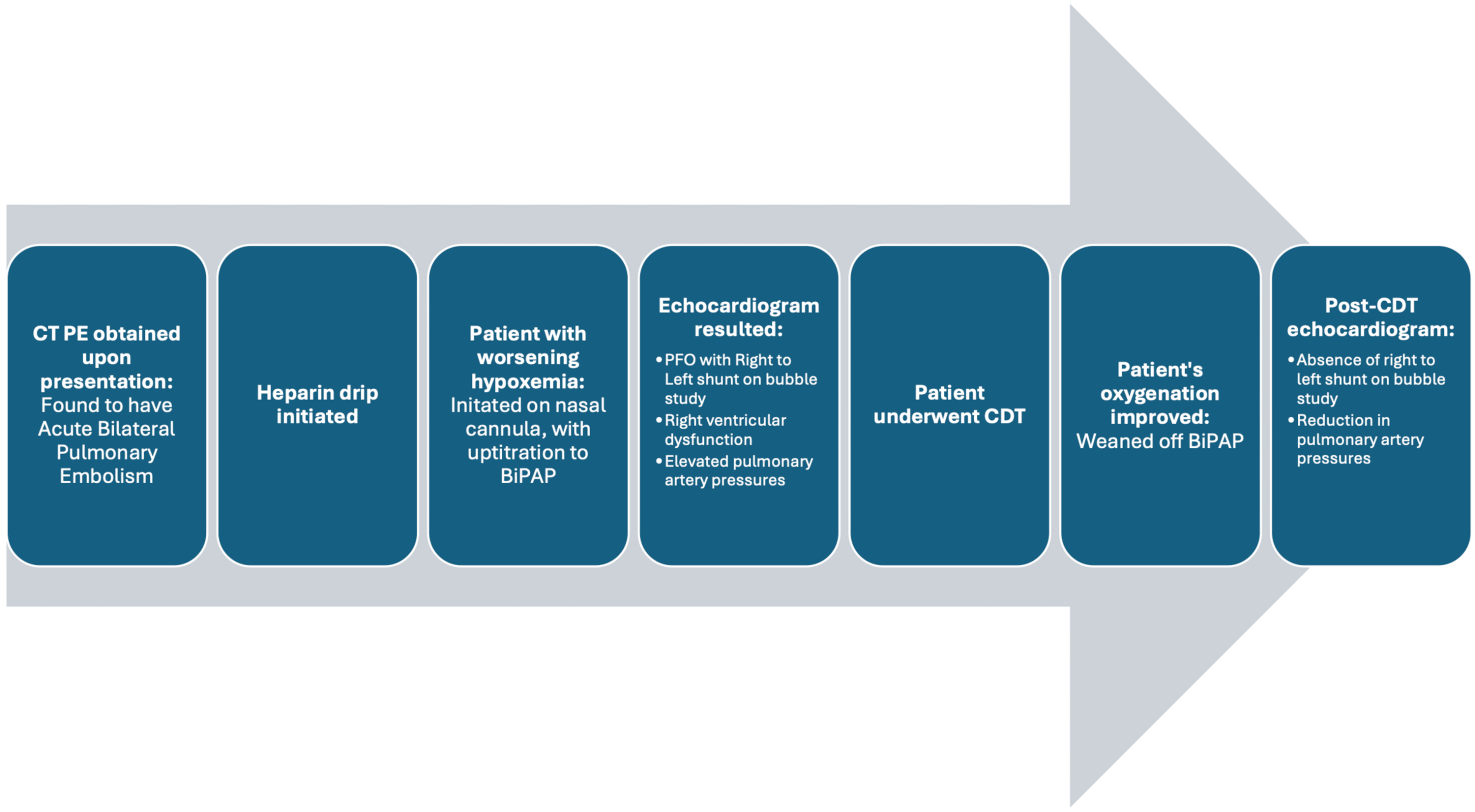
Timeline of events of clinical case presentation. PE: pulmonary embolism, CDT: catheter-directed thrombolysis, PFO: patent foramen ovale, BiPAP: bi-level positive airway pressure.

The patient was rapidly weaned off supplemental oxygen and subsequently discharged to an acute rehabilitation facility. The patient's clinical course is summarized in Figure 4.

## Discussion

While shortness of breath is a common presenting symptom of acute PE, up to 40% of patients with a new oxygen requirement have a normal arterial oxygen saturation [2]. Previous studies suggest that in patients with acute PE, the presence of a PFO is associated with a higher risk of mortality and an increased incidence of ischemic stroke [1,3,4]. In our case, the patient demonstrated refractory hypoxemia in which the patient continued to have worsening oxygenation despite increasing oxygen supplementation. Few cases have been reported in which patients with PFO presented with severe, refractory hypoxemia in the setting of acute PE [5].

In typical cases of PFOs, there is a left-to-right shunt, due to the increased pressures on the left side of the heart. However, acute PE acutely increases pulmonary vascular resistance (PVR), leading to elevated right-sided heart pressures, which can reverse the shunt direction from right to left [6]. This reversal allows deoxygenated blood to enter the systemic circulation from the right atrium to the left atrium, bypassing the lungs and resulting in hypoxemia [6]. In cases of acute PE without PFOs, significantly elevated pulmonary pressures can lead to right ventricular dysfunction [6]. In this instance, treatment or removal of the clot can reduce pulmonary pressures and alleviate right ventricular dysfunction. Similarly, given our patient’s intractable hypoxemia despite escalating supplemental oxygen, we recommended that reducing the PE clot burden would help lower pulmonary pressures, thereby reversing the shunt.

Of the previously reported cases of severe hypoxemia due to PFO in acute PE, 45% of patients survived [5]. A majority of these cases were treated with systemic thrombolysis (ST). Additional cases have been treated using unfractionated heparin, surgical embolectomy, or surgical closure of the PFO. Only one other case has been reported using CDT which was unsuccessful at treating the patient’s hypoxemia, and the patient ultimately had to undergo additional surgical embolectomy which resulted in resolution of the hypoxemia [5]. In our patient, hypoxemia persisted despite anticoagulation with a heparin drip but ultimately resolved following treatment with CDT. Additionally, post-CDT intervention, imaging demonstrated improvement in right heart function. While there have been minimal reports of CDT being used in the management of severe hypoxemia due to PFO in acute PE, studies have shown that CDT is beneficial over ST due to lower risks of bleeding in the treatment of general acute PE [7]. Prior to this case, there have been no reported instances of hypoxemia resolution or PFO closure in acute PE with CDT treatment. This case highlights the potential role of CDT in such scenarios; however, no studies have directly compared the outcomes of CDT with ST or surgical embolectomy.

Interestingly, a majority of reported cases of PFO in the setting of acute PE required mechanical ventilation due to the severity of hypoxemia [5]. In acute PE, supplemental oxygenation is recommended in patients when oxygen saturation (SpO_2_)is less than 90% [4]. In general, the administration of positive end-expiratory pressure (PEEP) improves oxygenation by recruiting areas of the atelectatic lungs; however, it also increases PVR and can worsen RV failure in acute PE [4,8]. A previous case report demonstrated that administration of PEEP via mechanical intubation can exacerbate right-to-left shunting, thereby worsening hypoxemia in cases of acute PE with PFO [8]. While our patient did not require intubation, she experienced persistent hypoxemia despite non-invasive positive pressure ventilation with BiPAP, suggesting that PEEP would not be beneficial in these cases. Rather, mechanical ventilation in patients with PFO in acute PE could lead to clinical decompensation and death. As such, in cases of refractory hypoxemia in patients with PFO in acute PE, administration of PEEP should be avoided, and intervention should instead be focused on alleviating clot burden in order to treat the hypoxemia.

## Conclusions

In cases of intractable hypoxemia associated with acute PE, the presence of an interatrial shunt should be considered. Treating the acute PE can reduce pulmonary pressures, potentially leading to shunt reversal or even PFO closure. CDT can be considered in the treatment in acute PE with PFO. Further studies are needed to compare outcomes between CDT, systemic thrombolysis, and surgical embolectomy in this high-risk patient population.
